# Maternal age 30–34 years and adverse perinatal outcomes: a systematic review and meta-analysis

**DOI:** 10.3389/fmed.2026.1845769

**Published:** 2026-07-01

**Authors:** Cai Xinna, Lu Xinhua, Nawsherwan, Yin Mei

**Affiliations:** 1 Department of Neonatology, Taixing People’s Hospital, Taizhou, Jiangsu, China; 2 School of Medicine, Fujian Branch of National Clinical Research Center for Cardiovascular Diseases, Xiamen Cardiovascular Hospital of Xiamen University, Xiamen, China

**Keywords:** congenital birth defects, intrauterine growth restriction, low birthweight, maternal age, perinatal mortality, preterm births, small-for-gestational age, stillbirth

## Abstract

**Background:**

Globally, births to women in their early thirties have become more prevalent. The current study aimed to examine the association of maternal age (30–34 years) with adverse perinatal outcomes, especially congenital birth defects and stillbirth.

**Methods:**

A literature search was conducted using PubMed, Web of Science, and Google Scholar for relevant studies published between July 1989 and August 2025. The primary outcomes were congenital birth defects (i.e., composite of Down syndrome and structural birth defects) and stillbirth. The secondary outcomes were preterm birth, low birthweight (LBW), neonatal mortality, small-for-gestational age (SGA), and intrauterine growth restriction (IUGR). Adverse perinatal outcomes were examined in women aged 30–34 compared with younger cohorts aged 18–29 years. The quality of the included studies and potential publication bias were evaluated. For the meta-analysis, forest plots were created for each outcome, and heterogeneity was assessed using the I^2^ statistic. Data were synthesized employing both random- and mixed-effects models.

**Results:**

Sixty studies were included in this meta-analysis. Maternal age (30–34 years) was associated with a significantly higher risk of overall congenital birth defects (OR, 1.10; 95% CI, 1.03, 1.17; *P* = 0.007; *I*^2^ = 92) compared with a reference age group of 18–29 years. The pooled subgroup analysis revealed that maternal age (30–34 years) showed a significant association with Down syndrome (OR, 1.97; 95% CI, 1.79, 2.16; *P* < 0.00001; *I*^2^ = 0) but a non-significant association with structural birth defects (OR, 0.99; 95% CI, 0.94, 1.05; *P* = 0.8; *I*^2^ = 84). Moreover, maternal age (30–34 years) showed a non-significant association with stillbirth (OR, 1.07; 95% CI, 0.94, 1.23; *P* = 0.3; *I*^2^ = 100), preterm birth, LBW, neonatal mortality, SGA, and IUGR.

**Conclusion:**

Maternal age 30–34 years was associated with a significantly higher risk of congenital birth defects, especially Down syndrome, but was not associated with other adverse perinatal outcomes, when compared to the 18–29 age group.

**Systematic review registration:**

https://www.crd.york.ac.uk/prospero/, identifier CRD420251105683.

## Introduction

Globally, progress in higher education and economic independence has empowered women to delay childbearing, leading to a significant rise in women choosing pregnancy after the age of 30 years (1, 2). Moreover, births to women aged 30 or older have increased substantially worldwide (3, 4). In the United States, the birth rate among women aged 30–34 years has increased from 21.1 to 26.6 per 1,000 women between 1990 and 2002 (5) and by 28% during 2000–2014 (6). In China, the proportion of births to women aged 30–34 years has increased by 32.5% between 2013 and 2017 (7). Similarly, the proportion of neonatal births to women aged 30 years or older increased dramatically across several European countries (8).

Maternal age (30–34 years) is associated with an increased risk of several adverse perinatal outcomes, including congenital birth defects (9, 10), stillbirth (11, 12), preterm birth (13, 14), neonatal death (11), low birthweight (LBW) (15, 16), and small for gestational age (SGA) (14). Although extensive research has examined adverse pregnancy outcomes in women with advanced maternal age ( ≥ 35 years) and younger age groups (17–19), the cohort of women aged 30–34 years remains understudied (20–22). This is a critical oversight, as women in this cohort represent a significant and growing proportion of global births, particularly in high-income countries where delayed childbearing is a common trend (6, 8). The precise risk of adverse perinatal outcomes in this cohort is poorly quantified and inconsistently documented in the literature. Prior research often categorized them within broader age groups 18–34 years (22, 23) or 25–34 years (20, 24), thereby masking the risk of adverse perinatal outcomes in this cohort. Recent studies suggest that even a 1-year increase within the 30s may increase the risk of adverse pregnancy outcomes (15, 16).

Several systematic reviews and meta-analyses determined the impact of advanced maternal age on adverse pregnancy outcomes (17, 18, 25–27) but failed to evaluate the association between maternal age (30–34 years) and adverse perinatal outcomes (17, 18, 25–27). Currently, no systematic review or meta-analysis has precisely quantified adverse perinatal outcomes for women aged 30–34 years. To address this gap, we conducted a systematic review and meta-analysis to synthesize the evidence for this cohort. Our findings aim to inform clinical counseling and public health guidelines, potentially establishing this group as distinct for tailored prenatal care.

## Methods

This systematic review and meta-analysis was performed in accordance with the Preferred Reporting Items for Systematic Review and Meta-Analysis (PRISMA) guidelines (28) and was prospectively registered and approved by the International Prospective Register of Systematic Review (PROSPERO) with registration ID: CRD420251105683. The use of publicly accessible data precluded the need for ethical approval or informed consent. The current systematic review and meta-analysis defined its key study questions using the PI(E)CO framework (i) population: pregnant women (ii) intervention or exposure: pregnant women aged 30–34 years (iii) controls: pregnant women aged 18–29 years (iv) outcomes: primary (congenital birth defects and stillbirth) and secondary (preterm birth, low birthweight (LBW), neonatal mortality, small-for-gestational-age (SGA), and intra-uterine growth restriction (IUGR).

### Definition of outcome variables

Congenital birth defects were defined as structural or chromosomal abnormalities that are present at birth and may affect the body’s development or appearance. The congenital birth defects were a composite of Down syndrome and structural birth defects. Down syndrome was defined as a chromosomal disorder caused by trisomy 21. Structural birth defects were defined as congenital abnormalities involving the structure or morphology of one or more body parts or organs that are present at birth and may affect appearance, function, or both. Stillbirth was defined as the birth of a fetus showing no signs of life following fetal death before or during delivery. Neonatal mortality was defined as the death of a live-born infant within the first 28 completed days of life. Preterm birth was defined as a neonate born before 37 completed weeks or fewer than 259 days from the first date of a woman’s last menstrual period. LBW was defined as birth weight < 2,500 g. SGA was defined as a newborn with birth weight below the 10th percentile for gestational age, based on standard sex-specific birth weight references. IUGR was defined as a pathological condition in which the fetus fails to achieve its genetically expected growth potential, commonly identified by estimated fetal weight or abdominal circumference below the 10th percentile for gestational age. One included article used the term fetal growth restriction (FGR) instead of IUGR. Because IUGR and FGR are frequently used interchangeably in the literature to describe impaired fetal growth, the article that reported FGR was included under the IUGR category for statistical analysis, while the original terminology reported in the article was retained during data extraction.

### Search strategy and selection process

We searched the electronic databases PubMed, Web of Science, and Google Scholar for relevant observational studies published between July 1989 and August 2025. The search strategy consisted of both Medical Subject Headings (MeSH) terms (e.g., “middle-aged,” “aged,” “maternal age,” “pregnancy outcome”) and key text words (e.g., “advanced maternal age,” “adverse perinatal outcomes”) in women aged 30–34 years. No time or geographic restrictions were applied, though the search was restricted to English-language publications. Two independent reviewers, CX and N assessed study eligibility by screening titles, abstracts, and full-text articles. Any discrepancies were resolved through consensus with a third researcher YM.

### Inclusion criteria

Studies were included if they met the following criteria: (1) involving a maternal age cohort of 30–34 years and observed its association with adverse perinatal outcomes; (2) compared adverse perinatal outcomes in this cohort (30–34 years) with a control group aged 18–24 or 18–29 years; (3) reported at least one primary or secondary outcome; and (4) provided clear numerator and denominator data for the study population and outcomes.

### Exclusion criteria

Studies were excluded for any of the following reasons: (1) the maternal age group of 30–34 years was missing or merged into a broad category; (2) the exact number of pregnant women and adverse perinatal outcome was not reported; (3) the study population consisted solely of twin pregnancies; (4) studies were not published in English; or (5) the publication type was a non-research article (e.g., case report, review, editorial).

### Data extraction

CX and LX extracted the data and recorded the following variables: first author, study year, study type, location, total population size, number of cases and controls, and outcome frequencies of each group. The extracted data were then verified by N.

### Assessment of bias risk

**FIGURE 1:**
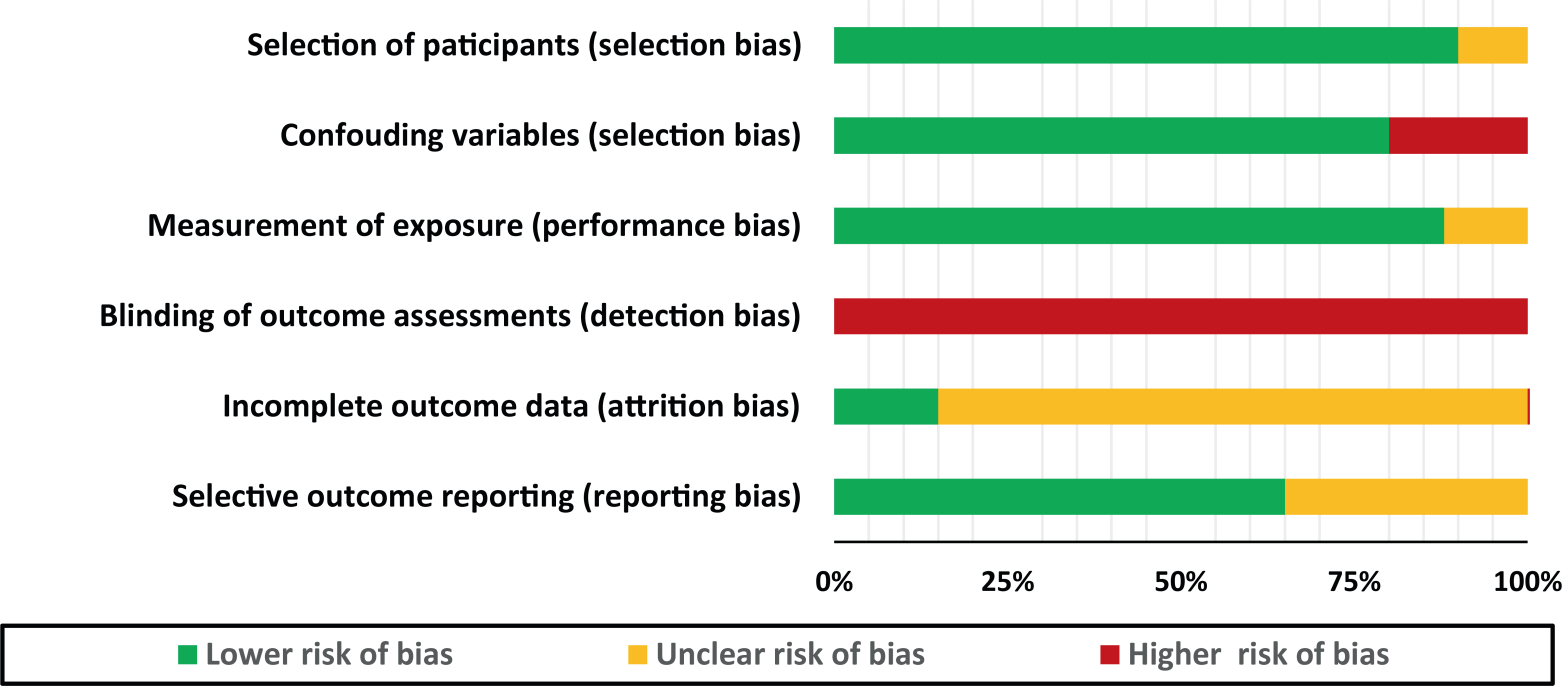
Risk of bias assessment for the included studies.

We employed the Risk of Bias Assessment Tool for Non-Randomized Studies (RoBANS) (29) to evaluate the quality of the included studies. As illustrated in Figure 1, the RoBANS tool assesses six key domains for potential bias:

1. Participant selection (selection bias)
2. Confounding variables (selection bias)
3. Measurement of exposure (performance bias)
4. Blinding of outcome assessments (detection bias)
5. Incomplete outcome data (attrition bias)
6. Selective outcome reporting (reporting bias)

Each domain was judged to have a low, high, or unclear risk of bias. The assessment was performed by two researchers (CX and YM). CX performed the initial evaluation, and YM reviewed and confirmed the ratings. The full evaluation criteria are available in Supplementary Table 1.

### Data synthesis and analysis

Primary outcomes (congenital birth defects and stillbirth) and secondary outcomes (preterm birth, LBW, neonatal mortality, SGA, and IUGR) were analyzed as dichotomous variables. We computed pooled odds ratios (ORs) with 95% confidence intervals (CIs). Heterogeneity was quantified with I*^2^* statistics and visualized using a forest plot. Given the significant heterogeneity (*I*^2^> 50%) and (*I*^2^< 50%), random-effects and fixed-effects models were used for the meta-analysis, respectively. To further investigate the results, we conducted subgroup analyses comparing the adverse perinatal outcomes in women aged 30–34 years against those aged 18–24 years. All tests were two-sided, and results were considered statistically significant at *P* < 0.05. The analyses were conducted using Review Manager software (version 5.4.1; Cochrane Collaboration, Copenhagen, Denmark).

## Results

### General characteristics of included studies

**FIGURE 2:**
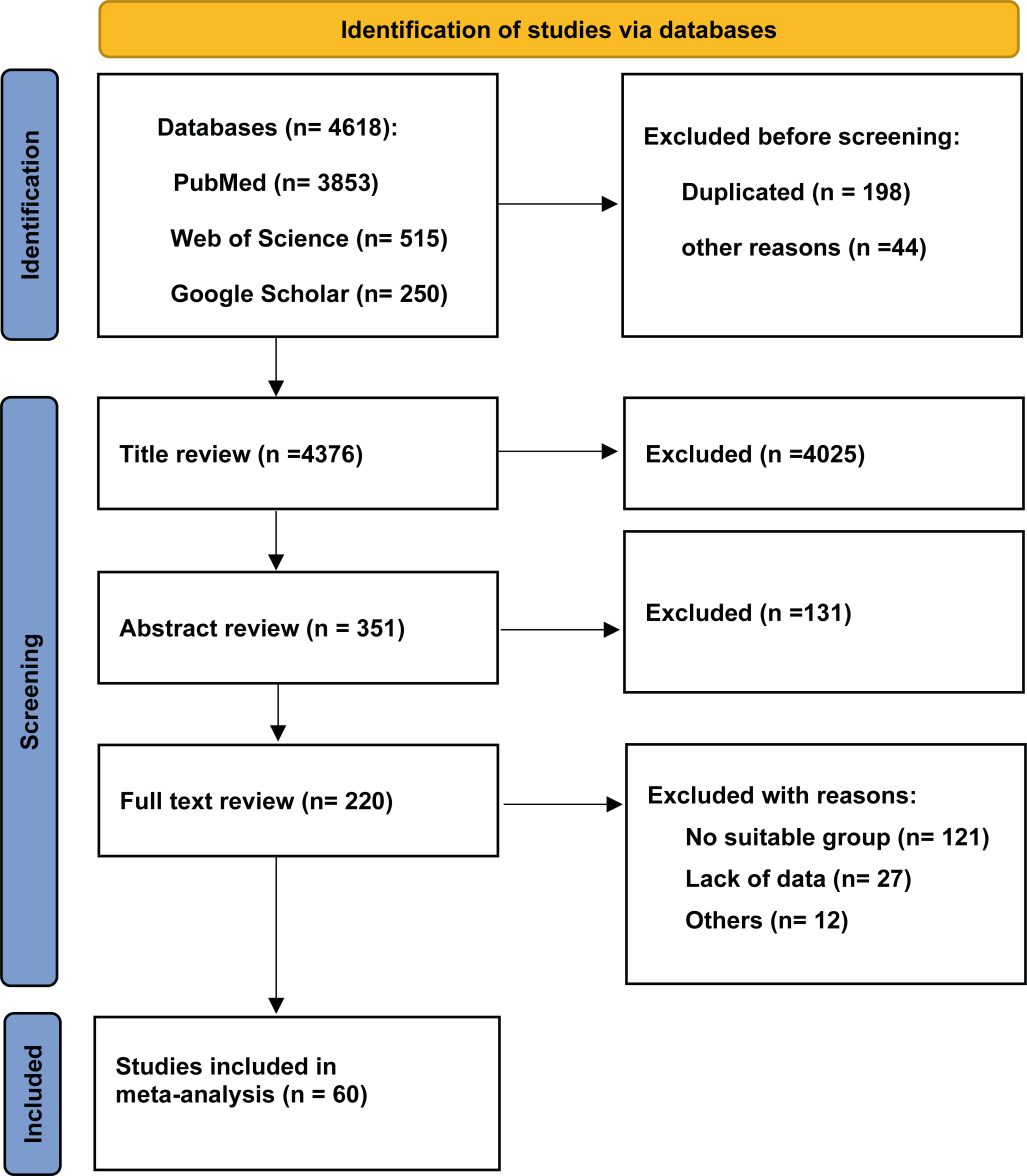
PRISMA flow diagram for systematic review and meta-analysis.

Figure 2 illustrates the search strategy and the process of study selection for the present systematic review and meta-analysis. A total of 60 studies were included in this review. The study consisted of over 120 million pregnant women, with 64.7 million aged 18–29 and 27.0 million aged 30–34. This systematic review and meta-analysis consisted of retrospective cohort studies (*n* = 51) (9–16, 30–72), cross-sectional studies (*n* = 7) (73–79), and prospective cohort studies (*n* = 2) (80, 81). This review presented data from 23 different populations. Most studies were from United States (*n* = 19) (12, 30, 31, 33, 43, 50, 53–57, 60–62, 64–66, 68, 70) followed by China (*n* = 11) (10, 15, 16, 45, 47, 48, 59, 71, 74, 77, 79), United Kingdom (*n* = 6)(32, 49, 51, 52, 73, 80), Sweden (*n* = 3) (14, 36, 38), Finland (*n* = 2) (37, 39), Canada (*n* = 2) (40, 44), Australia (*n* = 2) (58, 78), Spain (*n* = 2) (41, 76), and Thailand (*n* = 2) (9, 69). Single studies were reported from India (67), South Korea (13), Japan (75), Italy (46), Denmark (42), France (72), and Ghana (81). Moreover, four studies were from multi-national cohorts (11, 34, 35, 63) (Table 1).

**TABLE 1:** General characteristics of 31 included studies (*N* = 121,209,557).

Study	Study type	Year of study	Country	Total population (*N* = 121,209,557)	Women aged 18-24 years (*N* = 28,983,657)	Women aged 18–29 years (*N* = 64,738,970)	Women aged 30–34 years (*N* = 27,016,503)	Adverse perinatal outcomes
Stone et al. (49)	RCS	1989	UK	127,108	40,904	82,025	21,194	CBD (i.e., Down syndrome)
Martin et al. (50)	RCS	1990	USA	362,352	114,795	224,139	54,811	CBD (i.e., malformations of the aorta)
Lopez et al. (51)	RCS	1995	UK	141,784	NA	91,237	25,296	CBD (i.e., Down syndrome)
Carothers et al. (52)	RCS	1999	UK	323,779	75,641	192,187	78,351	CBD (i.e., Down syndrome)
Hollier et al. (53)	RCS	2000	USA	102,728	37,864	60,575	10,443	CBD (i.e., non-chromosomal fetal malformations)
Forrester et al. (54)	RCS	2002	USA	263,795	69,033	143,846	59,698	CBD (i.e., anal Atresia)
Forrester et al. (55)	RCS	2003	USA	258,350	65,389	1375,62	59,840	CBD (i.e., Down syndrome)
Forrester et al. (A) (56)	RCS	2004	USA	281,407	73,325	152,575	63,803	CBD (i.e., small intestinal atresia and stenosis)
Forrester et al. (B) (57)	RCS	2004	USA	250,935	73,325	152,575	63,803	CBD (i.e., Oral Clefts)
Vallino- Napoli et al. (58)	RCS	2004	Australia	1,120,907	200,983	605,719	331,171	CBD (i.e., Oral Clefts)
Canterino et al. (30)	RCS	2004	USA	21,610,873	5,440,685	11,441,496	4,970,770	Neonatal mortality
Carey et al. (78)	CSS	2005	Australia	1,223	290	753	262	CBD (i.e., isolated talipes equinovarus)
Tan et al. (59)	RCS	2005	China	328,096	34,835	150,151	117,733	CBD (birth defects)
Maconochie et al. (73)	CSS	2007	UK	6,719	NA	2,336	2,336	Stillbirth
Bahtiyar et al. (12)	RCS	2008	USA	6,239,399	1,775,850	3,266,844	617,385	Stillbirth
Salihu et al. (31)	RCS	2008	USA	1,313,677	429,647	871,365	265,167	Stillbirth
Salemi et al. (60)	RCS	2009	USA	1,179,418	299,118	607,347	262,762	CBD (i.e., Gastroschisis)
Glass et al. (61)	RCS	2009	USA	3,440,576	865,000	1,834,439	761,934	CBD (i.e., defects in the corpus callosum)
Koo et al. (13)	RCS	2012	South Korea	29,760	NA	7,950	15,496	CBD, stillbirth, preterm birth, LBW, SGA
Kenny et al. (32)	RCS	2013	UK	215,344	NA	122,307	62,371	Stillbirth, preterm birth, neonatal mortality, SGA,
Timofeev et al. (33)	RCS	2013	USA	203,517	51,011	107,491	45,715	Stillbirth, preterm birth, LBW, neonatal mortality
Luo et al. (79)	CSS	2013	China	12,630	4,324	7,874	2,469	CBD (i.e., CHD, Polydactyly, Equinovarus, Cleft Lip, Cleft Palate)
Winston et al. (62)	RCS	2014	USA	17,008	4,514	9,051	3,873	CBD (i.e., Hypospadias)
Blomberg et al. (14)	RCS	2014	Sweden	798, 674	185,942	486,764	205,905	Stillbirth, preterm birth, SGA
Li et al. (80)	PCS	2014	UK	63,371	11,395*	29,486	18,453	LBW
Waldenstrom et al. (34)	RCS	2014	Sweden, Norway	955,804	NA	526,545	319,057	Preterm birth, SGA, neonatal mortality
Xu et al. (74)	CCS	2014	China	1,860	NA	524	426	Stillbirth
Restrepo-Méndez et al. (35)	RCS	2015	Brazil, UK	20,242	4,742	110,17	4,701	Preterm birth, LBW
Waldenstrom et al. (36)	RCS	2015	Sweden	1,804,437	NA	602,681	573,810	Stillbirth
Bergman et al. (63)	RCS	2015	European countries	5,871,855	710,660	1,950,188	1,428,079	CBD (i.e., hypospadias)
Dawson et al. (64)	RCS	2015	USA	11,101	3,794	6,836	2,711	CBD (i.e., orofacial clefts)
Marshall et al. (65)	RCS	2015	USA	12,006,912	3,088,204	6,327,157	2,687,244	CBD (i.e., Omphalocele)
Mburia-Mwalili et al. (66)	RCS	2015	USA	124,341	26,729	66,333	32,504	CBD (i.e., birth defects)
Bhat et al. (67)	RCS	2016	India	20,432	4,123	15,381	4,602	CBD (i.e., congenital urogenital anomalies)
Cragan et al. (68)	RCS	2016	USA	11,110,665	2,608,005	5,750,710	2,765,901	CBD (i.e., microcephaly)
Jaruratanasirikul et al. (69)	RCS	2016	Thailand	186,393	47,073	95,535	39,702	CBD (i.e., Oral cleft)
Klemetti et al. (37)	RCS	2016	Finland	228,348	56,282	142,822	60,716	Preterm birth, LBW, neonatal mortality
Yoshioka-Maeda et al. (75)	CSS	2016	Japan	3,245	NA	702	677	Preterm birth, LBW, IUGR,
Jaruratanasirikul et al. (9)	RCS	2017	Thailand	186,393	69,338	117,800	39,702	CBD (i.e., Down syndrome)
Louis et al. (70)	RCS	2017	USA	13,108,466	3,136,617	6,615,611	3,145,042	CBD (i.e., birth defects)
Waldenstrom et al. (38)	RCS	2017	Sweden	2,009,068	343,560	1,053,756	646,736	Preterm birth
Goisis et al. (39)	RCS	2017	Finland	124,098	20,562	66,508	37,580	Preterm birth, LBW
Fuchs et al. (40)	RCS	2018	Canada	165,282	24,650	83,774	55,867	Preterm birth
Odame Anto et al. (81)	PCS	2018	Ghana	175	NA	62	55	Stillbirth, preterm birth, LBW, IUGR
Xie et al. (71)	RCS	2018	China	6,73,060	188,786	482,922	125,233	CBD (i.e., CHD)
Bruckner et al. (72)	RCS	2019	France	849,094	104,392	340,673	292,058	CBD (i.e., Down syndrome)
Yi et al. (10)	RCS	2019	China	13,284,142	3,339,333	8,920,907	2,856,847	CBD (i.e., holoprosencephaly)
Casteleiro et al. (76)	CSS	2019	Spain	3,315	NA	507	1,582#	Stillbirth, neonatal mortality, LBW, SGA,
Claramonte Nieto et al. (41)	RCS	2019	Spain	25,054	NA	2,437	9,643	Stillbirth, preterm birth
Rydahl et al. (42)	RCS	2019	Denmark	1,122,964	NA	517,450	398,873	Preterm birth, LBW, neonatal mortality
Nguyen et al. (43)	RCS	2019	USA	12,710	NA	3,143	1,840	Stillbirth
Schummers et al. (44)	RCS	2019	Canada	16,514,8 49	4,919,066	9,151,351	2,834,401	Stillbirth, preterm birth, SGA, neonatal mortality
Bi et al. (77)	CSS	2021	China	10,206	2,711	8,235	1,751	FGR, preterm
Cao et al. (45)	RCS	2022	China	583,571	121,622	400,941	128,533	Preterm birth, SGA
Esposito et al. (46)	RCS	2022	Italy	741,150	40,806	184,139	272,934	Preterm birth
Hou et al. (47)	RCS	2022	China	7,715	147	1,382	3,297	Preterm birth, LBW, SGA
Nawsherwan et al. (48)	RCS	2022	China	22,950	NA	11,282	7,732	CBD, preterm birth, LBW, neonatal mortality, IUGR
Huang et al. (16)	RCS	2023	China	63,137	9,872	41,736	15,834	Stillbirth, preterm birth, SGA, LBW
Li et al. (15)	RCS	2023	China	60,209	13,424	40,957	13,041	CBD (i.e., microtia), stillbirth, preterm birth, LBW, SGA,
Nyongesa et al. (11)	RCS	2023	Democratic Republic of Congo, Guatemala, India, Kenya, Pakistan, Zambia	602,884	245,289	408,872	86,751	Stillbirth, preterm birth, LBW, neonatal mortality

RCS, Retrospective cohort study; PCS, prospective cohort study; CSS, cross-sectional study; NA, not available; CBD, congenital birth defects; LBW, low birthweight; SGA, small-for-gestational age; IUGR, intrauterine growth restriction; FGR, fetal growth restriction.

### Risk of bias assessment

**FIGURE 3:**
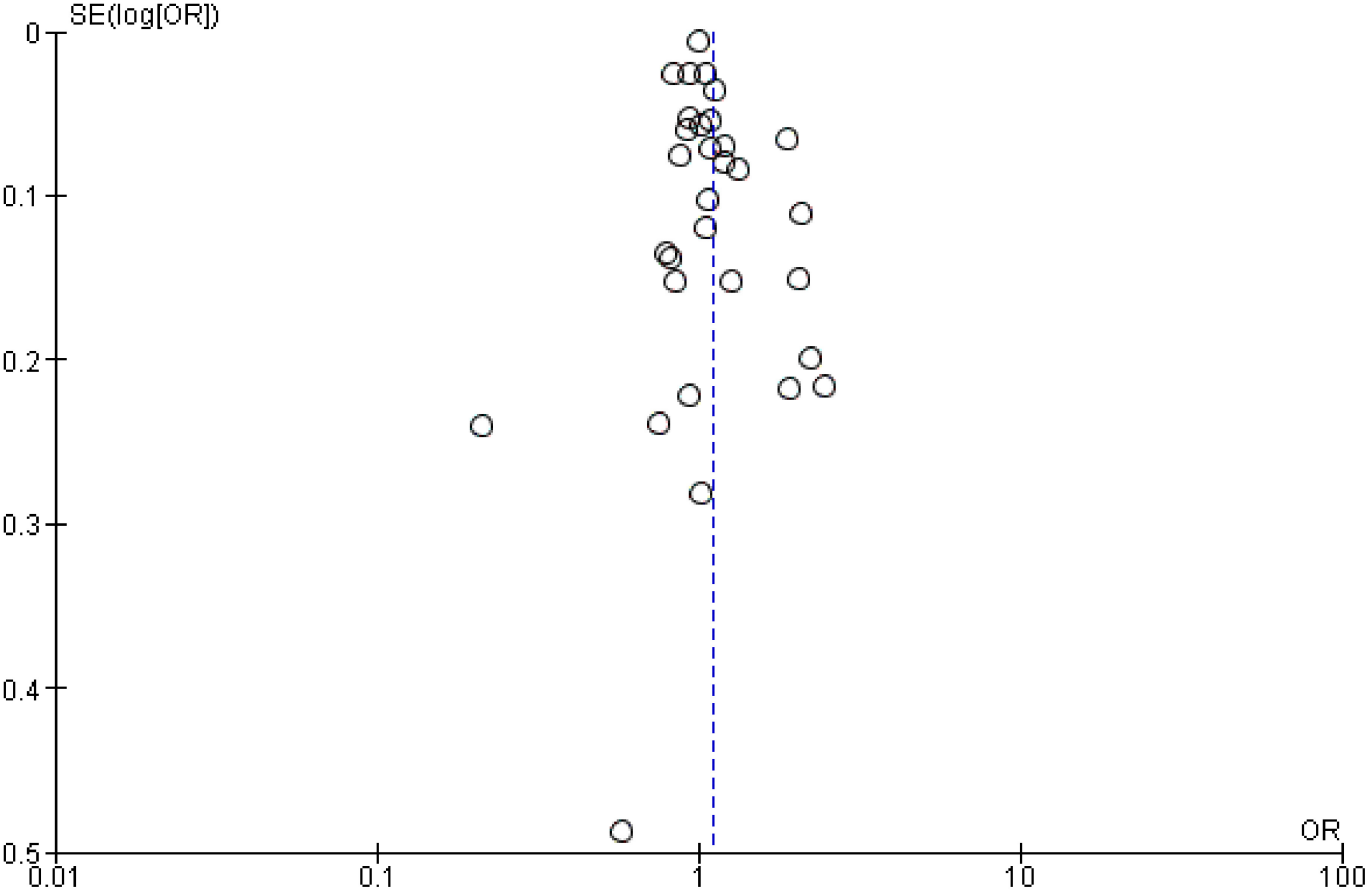
Funnel plot for publication bias.

Figure 1 presents the risk of bias assessment using the RoBANS tool. A lower risk of bias was identified in over half of the studies across the domains of selection of participants, confounding variables, measurement of exposure, and selective outcome reporting. However, a higher risk of bias was demonstrated in the majority of the studies for the blinding of outcome assessments, while the risk for incomplete outcome data was largely unclear. Furthermore, a visual inspection of the funnel plot suggested no publication bias (Figure 3).

### Primary outcomes

**FIGURE 4:**
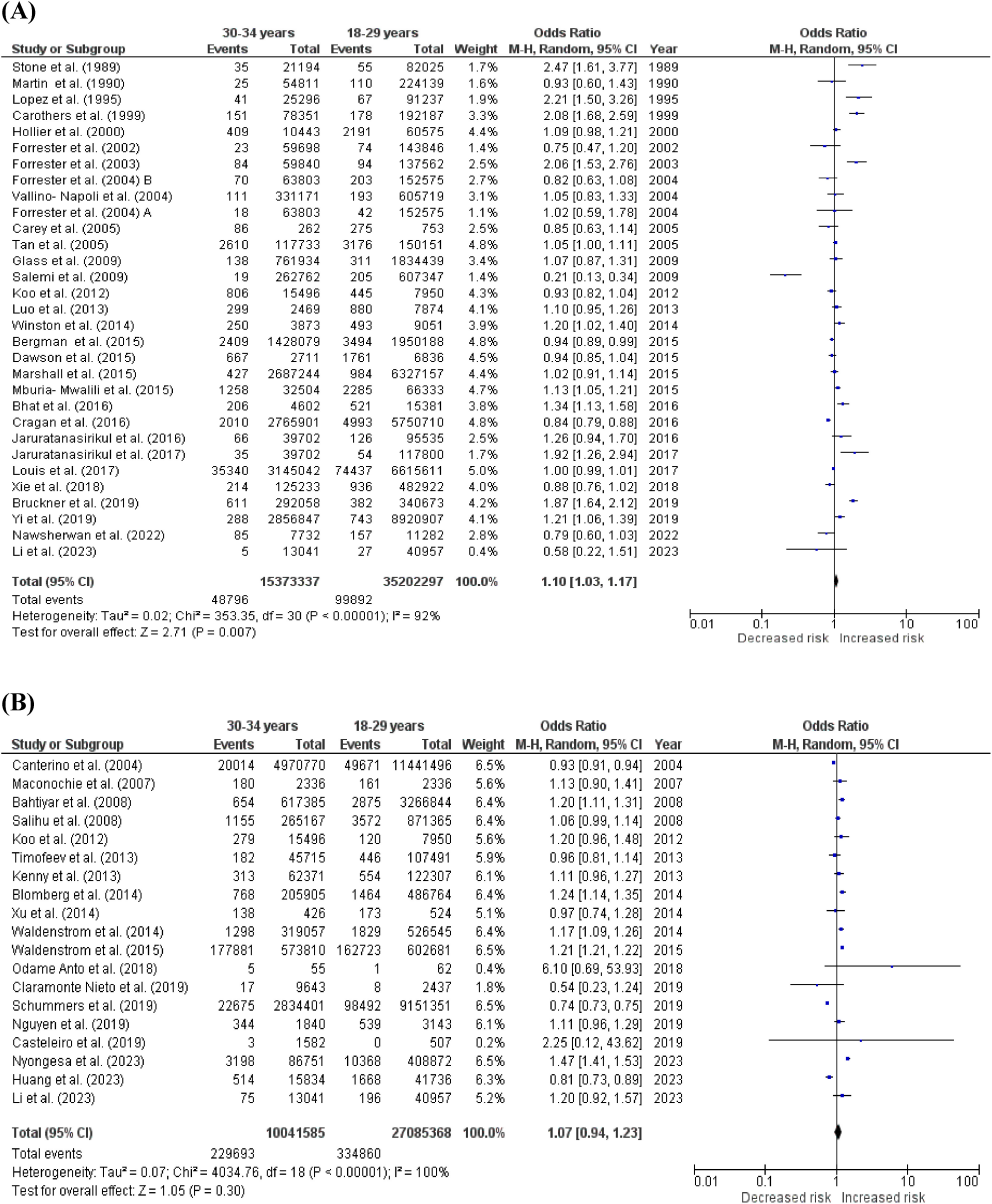
Forest plot of odds ratios of overall congenital birth defects **(A)** and stillbirth **(B)** in babies of women aged 30–34 years compared with women aged 18–29 years.

The primary outcomes were congenital birth defects and stillbirth. The congenital birth defects were a composite of Down syndrome and structural birth defects. A total of 31 studies examined the risk of congenital birth defects, including Down syndrome (9, 49, 51, 52, 55, 72) and structural birth defects (10, 13, 15, 48, 50, 53, 54, 56–71, 78, 79) in women aged 30–34 years. We found that maternal age (30–34 years) was associated with a significantly higher risk of overall congenital birth defects (31 studies; OR, 1.10; 95% CI, 1.03, 1.17; *P* = 0.007; *I*^2^ = 92) compared with a reference age group 18–29 years (Figure 4A).

**FIGURE 5:**
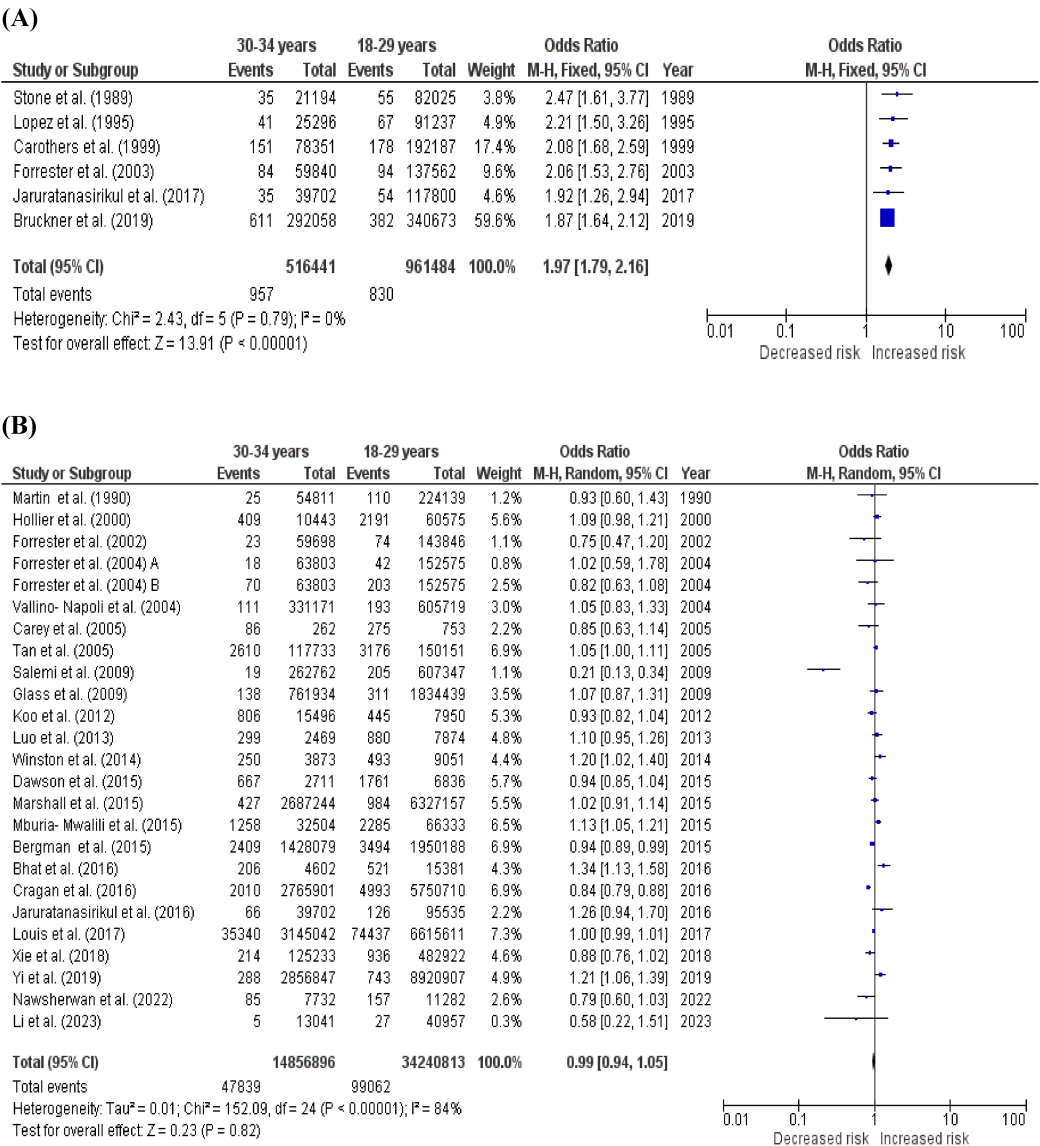
Forest plot of odds ratios of chromosomal defects **(A)**, and structural birth defects **(B)** in babies of women aged 30–34 years compared with women aged 18–29 years.

Further, the subgroup analysis by types of congenital birth defects, maternal age (30–34 years) showed a significant association with Down syndrome (6 studies; OR, 1.97; 95% CI, 1.79, 2.16; *P* < 0.00001; *I*^2^ = 0) but non-significant association with structural birth defects (25 studies; OR, 0.99; 95% CI, 0.94, 1.05; *P* = 0.8; *I*^2^ = 84) (Figures 5A,B). Moreover, in subgroup analysis when compared with a young cohort (18–24 years), maternal age (30–34 years) observed a significant association with overall congenital birth defects (28 studies; OR, 1.12; 95% CI, 1.02, 1.22; *P* = 0.01; *I*^2^ = 93) and Down syndrome (5 studies; OR, 2.29; 95% CI, 1.98, 2.64; *P* < 0.00001; *I*^2^ = 0) but non-significant association with structural birth defects (23 studies; OR, 1.00; 95% CI, 0.92, 1.08; *P* = 0.9; *I*^2^ = 91) (Supplementary Figures 1A, 2A,B).

The pooled analysis (*n* = 19) showed no statistically significant association between maternal age 30–34 years and the risk of stillbirth compared with women aged 18–29 years (OR, 1.07; 95% CI, 0.94, 1.23; *P* = 0.3; *I*^2^ = 100). However, 5 studies individually reported that women aged 30–34 years were significantly associated with a higher risk of stillbirth with odds ratios ranging from 1.20 to 1.47 (11, 12, 14, 34, 36) (Figure 4B). Moreover, the pooled subgroup analyses showed that women aged 30–34 years were not significantly associated with stillbirth (9 studies; OR, 1.07; 95% CI, 0.92, 1.24; *P* = 0.3; *I*^2^ = 99) when compared with young women aged 18–24 years (Supplementary Figure 1B).

### Secondary outcomes

**FIGURE 6:**
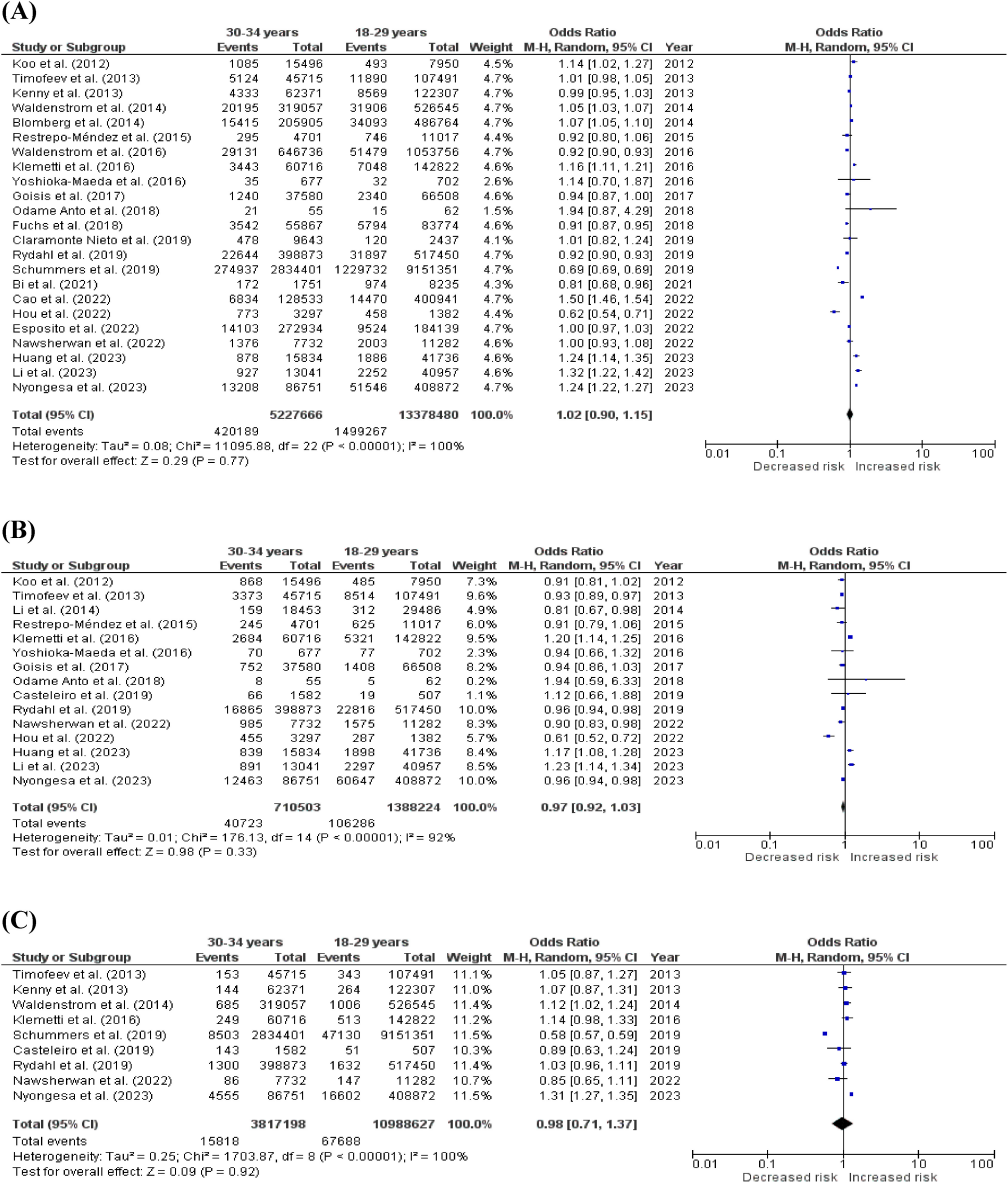
Forest plot of odds ratios for preterm births **(A)**, LBW **(B)**, and neonatal mortality **(C)** among women aged 30–34 years compared with women aged 18–29 years.

The secondary outcomes were preterm birth, LBW, neonatal mortality, SGA, and IUGR. A total of 23 studies examined the risk of preterm births in women aged 30–34 years. Among these, 8 studies reported a higher risk (11, 13–16, 34, 37, 45), while the majority (*n* = 15) (32, 33, 35, 38–42, 44, 46–48, 75, 77, 81) showed no significant association between maternal age (30–34 years) and preterm births compared with a reference age group 18–29 years. Across these eight studies, the odds ratios of preterm birth in women aged 30–34 years ranged from 1.05 to 1.50. A pooled analysis of 23 studies (11, 13–16, 32–35, 37–42, 44–48, 75, 77, 81) showed a non-significant odds ratio for preterm birth in women aged 30–34 years (OR, 1.02; 95% CI, 0.90, 1.15; *P* = 0.7; *I*^2^ = 100) compared with women aged 18–29 years. However, considerable heterogeneity was observed (*I*^2^ = 100), suggesting the results are not consistent across studies and should be interpreted with caution (Figure 6A).

**FIGURE 7:**
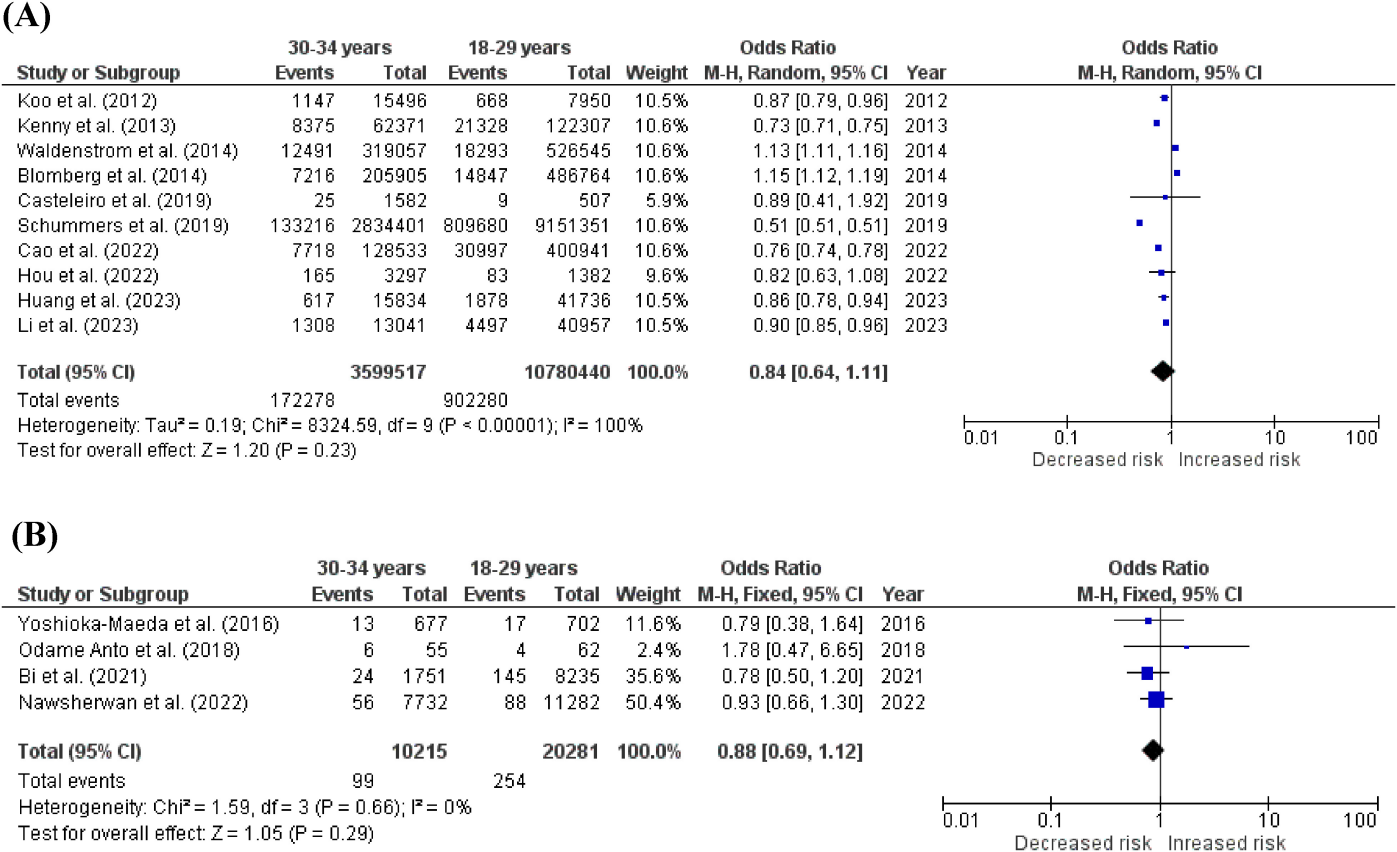
Forest plot of odds ratios for SGA **(A)** and IUGR **(B)** among women aged 30-34 years compared with women aged 18-29 years.

The pooled analyses indicate that women aged 30–34 years were not significantly associated with LBW (15 studies; OR, 0.97; 95% CI, 0.92, 1.03; *P* = 0.3; *I*^2^ = 92), neonatal mortality (9 studies; OR, 0.98; 95% CI, 0.71, 1.37; *P* = 0.9; *I*^2^ = 100), SGA (10 studies; OR, 0.84; 95% CI, 0.64, 1.11; *P* = 0.2; *I*^2^ = 100), and IUGR (4 studies; OR, 0.88; 95% CI, 0.69, 1.12; *P* = 0.2; *I*^2^ = 0) compared with women aged 18–29 years (Figures 6B,C, 7A,B).

Furthermore, the pooled subgroup analyses revealed a non-significant association between maternal age (30–34 years), preterm births, LBW, and neonatal mortality compared with young women aged 18–24 years. However, women aged 30–34 years showed significantly lower risk of SGA (6 studies; OR, 0.79; 95% CI, 0.66, 0.94; *P* = 0.009; *I*^2^ = 99) compared with the young cohort 18–24 years (Supplementary Figures 3A–D).

## Discussion

The current systematic review and meta-analysis, which consisted of over 120 million births, was conducted to explore the association between maternal age 30–34 years and adverse perinatal outcomes, including congenital birth defects, stillbirth, preterm births, LBW, perinatal mortality, SGA, and IUGR, compared with 18–29 years. This meta-analysis revealed a significantly higher risk of overall congenital birth defects in women aged 30–34 years. Further, the subgroup analysis showed that maternal age (30–34 years) was significantly associated with Down syndrome but non-significantly associated with structural birth defects. Moreover, maternal age (30–34 years) showed a non-significant association with stillbirth, preterm births, LBW, neonatal mortality, SGA, and IUGR.

### Primary outcomes

This systematic review meta-analysis found a significantly higher risk of overall congenital birth defects in women aged 30–34 years than in the maternal age group 18–29 years. Furthermore, as a result of the subgroup analysis by types of congenital birth defects, maternal age (30–34 years) was observed to have a significant association with Down syndrome but a non-significant association with structural birth defects. The significant association between maternal age 30–34 years and Down syndrome was consistent across all six included individual studies (9, 49, 51, 52, 55, 72). Moreover, the majority of the included studies showed a non-significant association between maternal age 30–34 years and structural birth defects. However, five studies showed the opposite findings (10, 59, 62, 66, 67). Although the pooled association for overall congenital defects was modest, this composite outcome included both Down syndrome and structural birth defects. Notably, the analysis showed a stronger association for Down syndrome; therefore, the overall estimate should be interpreted cautiously in relation to the separate findings for Down syndrome and structural birth defects.

The predominant risk factor for Down syndrome is increasing maternal age, especially in women aged ≥ 35 years (55). However, this meta-analysis showed that even women aged 30–34 years are at a higher risk of having babies with Down syndrome. These findings should be interpreted as associations rather than causal effects because most included studies were observational. Previous systematic review meta-analysis observed that young maternal age ( < 20 years old) and advanced maternal age ( ≥ 35 years) are associated with a higher risk of congenital anomaly compared with women aged 20–34 years. Furthermore, they examined a higher risk of chromosomal anomaly in women aged ≥ 35 years but a lower risk in young maternal age ( < 20 years old) (27). Several studies investigated the age-specific incidence rate of Down syndrome by single-year intervals and found that maternal age was positively associated with the incidence of Down syndrome. The rate was significantly higher among women aged 30–34 years (1.15–2.29 per 1,000 births) compared to women aged 20–29 years (0.65–0.84 per 1,000 births) (82).

Apart from maternal age, errors in sister chromatid segregation and a reduction in chromosome cohesion are key biological mechanisms contributing to chromosomal abnormalities in oocytes. It has been suggested that in older women, chromosomal anomaly in offspring could be due to telomere shortening and oxidative stress in oocytes, and non-chromosomal anomaly may be attributed to cumulative teratogen exposure, environmental toxins, and medical issues (e.g., gestational diabetes). Since these anomalies can cause severe disability and reduce fetal survival, preventive strategies are a critical public health priority (27).

In this systematic review and meta-analysis, we found that women aged 30–34 years experienced a marginally higher risk of stillbirth, but this was not statistically significant. However, some studies individually reported that maternal age (30–34 years) was significantly associated with a higher risk of stillbirth (11, 12, 14, 34, 36). A population-based study (36) and a multi-national cohort-based study reported a linear association between maternal age and the risk of stillbirth (11). This systematic review and meta-analysis is the first to quantify the association between maternal age 30–34 years and the risk of stillbirth, addressing previously inconsistent findings in the literature. Prior systematic review and meta-analysis revealed a higher risk of stillbirth among women with advanced maternal age ( ≥ 35 years or ≥ 40 years) (17, 26). Placental aging and insufficiency are the predominant explanations for the association between advanced maternal age and stillbirth. With increasing age, sclerotic lesions can cause placental underperfusion and impair nutrient flux to the fetus, which may lead to intrauterine fetal demise and stillbirth (36, 83).

### Secondary outcomes

Preterm births, LBW, neonatal mortality, SGA, and IUGR were investigated in this systematic review and meta-analysis. This meta-analysis showed a slightly higher risk of preterm birth in women aged 30–34 years compared to those aged 18–29 years; however, this association was not statistically significant. This non-significant association was consistent across the 15 included studies (32, 33, 35, 38–42, 44, 46–48, 75, 77, 81). In contrast, 8 studies individually reported a higher risk of preterm birth among women aged 30–34 years (11, 13–16, 34, 37, 45). Previous systematic review and meta-analysis reported that advanced maternal age ( ≥ 35 years or ≥ 40 years) is associated with a higher risk of preterm birth (17, 26). However, research shows that the risk of preterm births starts to increase before women reach the traditional cut-off age of 35 years (11, 13–16, 34, 37, 45). These findings suggest that maternal age has a linear effect on preterm birth rather than a threshold effect (84). Therefore, establishing rigid cutoff points, such as 35 or 40 years, for advanced maternal age is not well-supported (17, 85). These findings suggest that women below the threshold for “advanced maternal age” also require focused care. Increasing antenatal surveillance and health education for this group could lead to improved pregnancy outcomes.

The pooled analysis indicated that women aged 30–34 years were not significantly associated with LBW, neonatal mortality, SGA, and IUGR. However, several individual studies reported a significantly higher risk of LBW (15, 16, 37), neonatal mortality (11, 34), and SGA (14, 34) in women aged 30–34 years. These contradictory findings could be attributed to factors such as study setting (e.g., population-based vs. hospital-based), resource availability, and variation in the prevalence of obstetric complications, parity, and socioeconomic status among the studied populations (11, 15, 44, 47).

### Subgroup analysis

Subgroup analysis by age group revealed that women aged 30–34 years showed an increased likelihood of having babies with overall congenital birth defects, particularly Down syndrome, compared to women aged 18–29 years. This association was even more pronounced when comparing them to the lowest-risk age group, 18–24 years, indicating that the measured risk is highly sensitive to the age group used for comparison. Our findings highlight a critical gap: despite the known increased risk of adverse perinatal outcomes for maternal age (30–34 years), no national guidelines exist for this age cohort. To address this, future research must use more precise age categories to quantify these risks accurately. We recommend implementing targeted care, such as increased prenatal monitoring, enhanced congenital birth defects screening, and multidisciplinary support. It is also essential to educate this group about their specific risks to ensure they receive appropriate healthcare.

### Strengths and limitations

The principal strength of this systematic review and meta-analysis lies in its novel focus on the 30–34 years’ maternal age cohort and the large sample size ( > 120 million births), which provides a robust statistical power. Several limitations are also noted. Primarily, inconsistent assessment and categorization of maternal age across studies may have introduced misclassification bias, while very high heterogeneity may limit the reliability of publication bias assessment. Although 18–29 years was used as the primary reference maternal age group, some included studies used narrower reference categories such as 18–24 years. This inconsistency in age categorization may have introduced misclassification bias and may partly explain the observed heterogeneity; therefore, the findings should be interpreted with caution. Moreover, the analyses revealed significant heterogeneity, which is likely explained by contextual differences across the studies’ geographic, ideological, and medical settings, in addition to variations in sample sizes and diagnostic criteria. Furthermore, because most included studies were observational, the pooled findings should be interpreted as associations rather than causal effects. Although we used the most fully adjusted estimates where available, adjustment for confounders varied across studies, and some studies did not provide adjusted estimates. Therefore, residual confounding cannot be excluded, particularly for factors such as maternal body mass index, diet, lifestyle, parity, pre-existing conditions, socioeconomic status, and access to antenatal care.

## Conclusion

This systematic review and meta-analysis observed that women aged 30–34 showed a significantly increased likelihood of having babies with congenital birth defects, especially Down syndrome, compared with young women. In addition, maternal age (30–34 years) showed a non-significant association with stillbirth, preterm births, LBW, neonatal mortality, SGA, and IUGR. Based on these results, future clinical guidelines should consider women aged 30–34 years as a distinct group that may benefit from enhanced counseling and screening for congenital birth defects, particularly Down syndrome. Future research with large sample sizes is needed to determine the relationship with stillbirth conclusively.

## Data Availability

The original contributions presented in this study are included in the article/Supplementary material, further inquiries can be directed to the corresponding authors.
