# Supplementary material for: Maternal age 30–34 years and adverse perinatal outcomes: a systematic review and meta-analysis
Source: Front Med (Lausanne). 2026 Jul 1;13:1845769. doi: 10.3389/fmed.2026.1845769 (PMC13369265; doi:10.3389/fmed.2026.1845769)
Supplement: Supplementary file 1 [file Data_Sheet_1.docx]

**(A)**

**
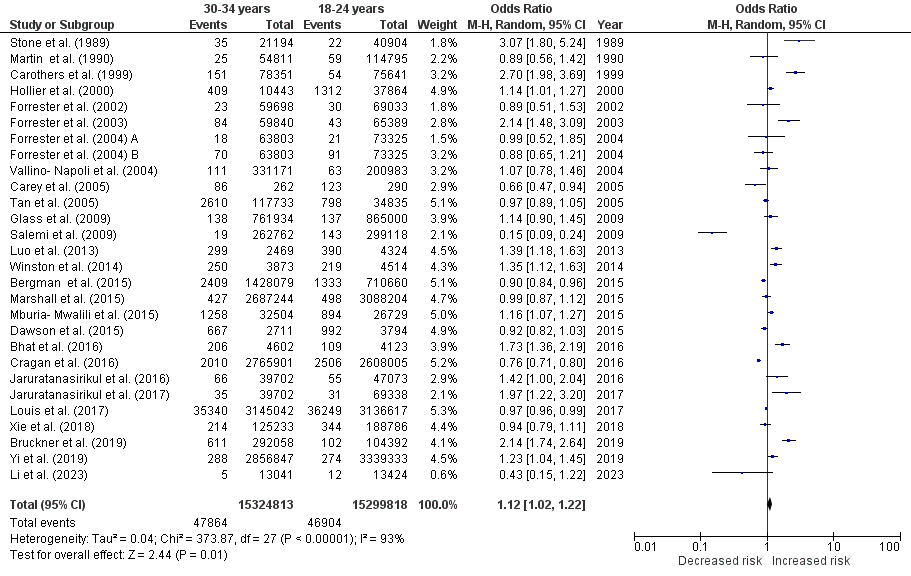
**

**(B)**

**
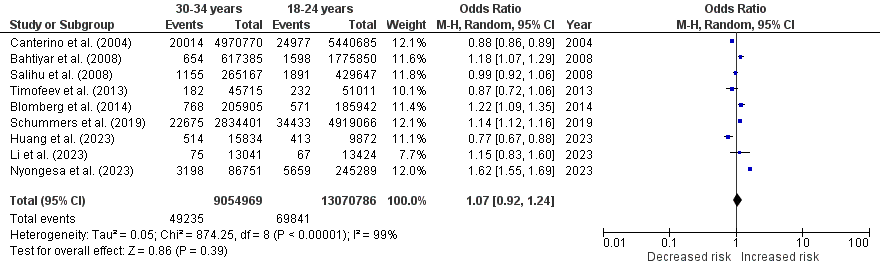
**

**Figure S1:** Forest plot of odds ratios of overall congenital birth defects (A) and stillbirth (B) in babies of women aged 30-34 years compared with women aged 18-24 years

**(A)**

**
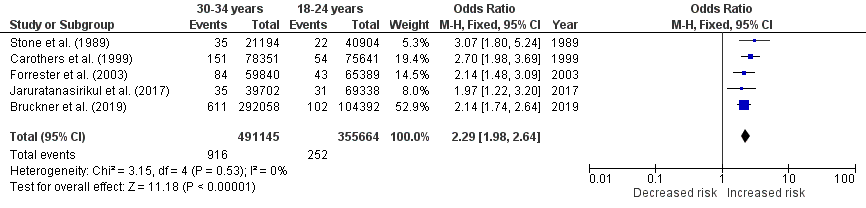
**

**(B)**

**
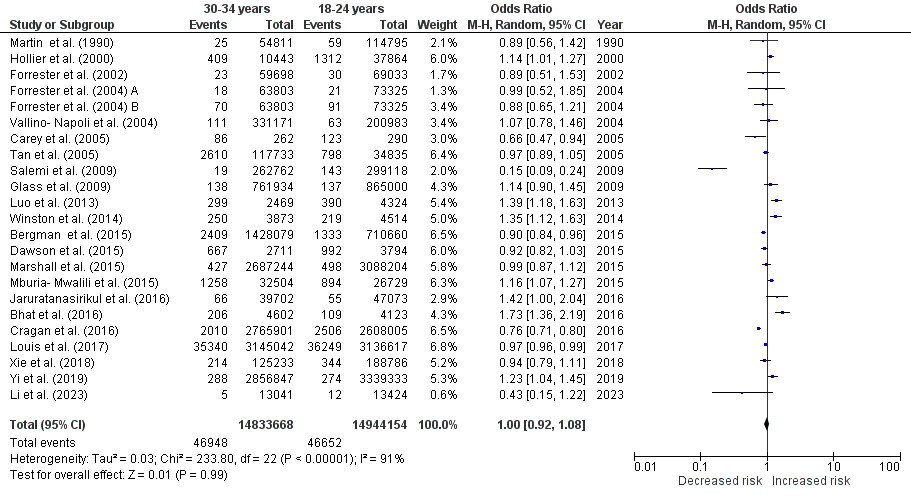
**

**Figure S2:** Forest plot of odds ratios of chromosomal defects (A), and structural birth defects (B) in babies of women aged 30-34 years compared with women aged 18-24 years

**(A)**

**
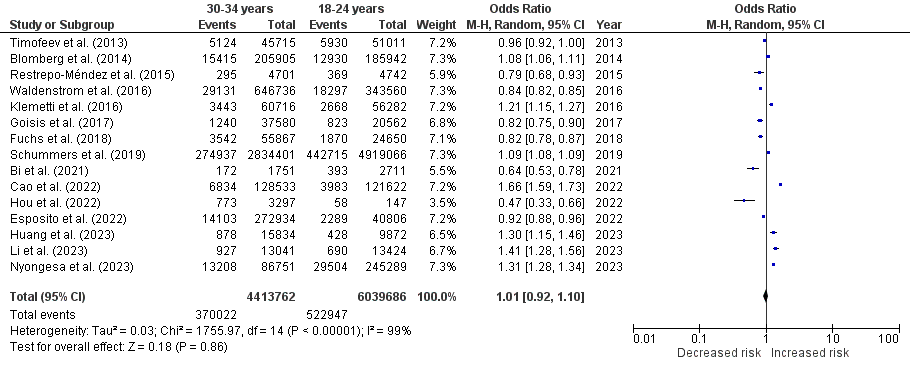
**

**(B)**

**
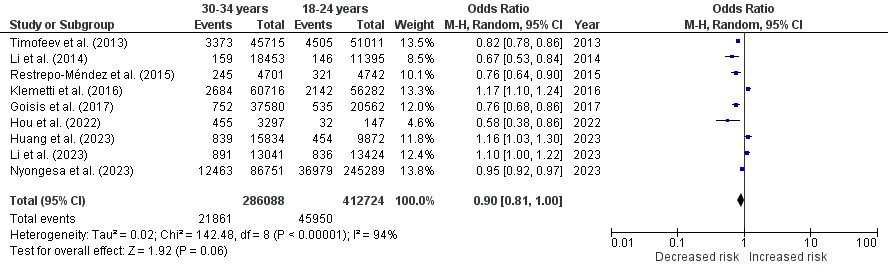
**

**(C)**

**
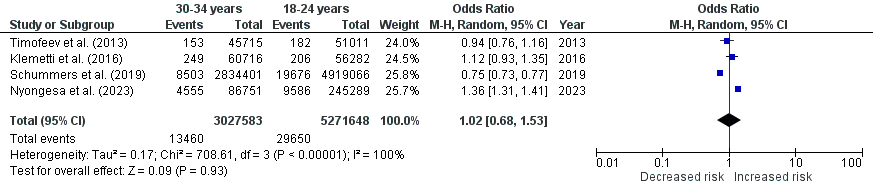
**

**(D)**

**
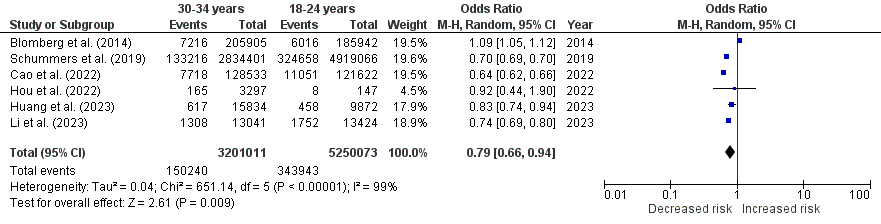
**

**Figure S3:** Forest plot of odds ratios for preterm births (A), LBW (B), neonatal mortality (C), and SGA (D) among women aged 30-34 years compared with women aged 18-24 years

**Table S1:** Criteria for evaluation of the Risk-of-Bias Assessment Tool for Non-Randomized Studies (RoBANS) tool.

| 1. The selection of participants  Selection biases caused by the inadequate selection of participants | |
| --- | --- |
| Criteria for judgments of a 'Low risk' of bias | **Cohort study, Non-randomized controlled trial**  Intervention (exposure) and control groups are the same population group (identical institution and period), and the absence of outcomes among the study participants was confirmed at the starting point of the study.  **Case-control study**  The case and control groups were selected from comparable population groups. The case group was clearly defined, and it was clearly demonstrated that the control group is not the patient group.  **Before-after study**  The study participants were consecutively recruited, and the data were collected prospectively. |
| Criteria for judgments of a 'High risk' of bias | Any one of the following conditions:  **Cohort study, Non-randomized controlled trial**  ○ The intervention (exposure) and control groups were selected from different population groups (e.g., the intervention group differs from the control group with respect to study period or study center, or historical  control groups were used).  ○ The presence of outcomes among the study participants was not confirmed at the starting point of the study.  **Case-control study**  ○The case and control groups are not the comparable population groups.  ○ The patient definitions were generated by self-reported or merged data.  ○ It was not clearly confirmed that the control group excluded patients.  **Before-after study**  ○ The control group was not recruited consecutively.  ○ Retrospective data collection was performed. |
| Criteria for judgments of an 'Unclear risk' of bias | It is uncertain whether the incomplete outcome data resulted in a 'high risk' or a 'low risk' of bias. |
| 2. Confounding variables  Selection biases caused by the inadequate confirmation and consideration of confounding variables | |
| Criteria for judgments of a 'Low risk' of bias | Any one of the following conditions:  **Non-randomized studies (except for before-after studies)**  ○ The major confounding variables were adequately confirmed and considered during the design phase (e.g., through matching, participation restriction, or other methods).  ○ The major confounding variables were adequately confirmed and adjusted for during the analysis phase (e.g., through stratification, propensity score approaches, statistical adjustments, or other methods).  **Before-after study**  ○ A natural progression and learning effect* can be excluded during the consideration of diseases and interventions. |
| Criteria for judgments of a 'High risk' of bias | Any one of the following conditions:  **Non-randomized study (except for before-after studies)**  ○ The major confounding variables were not considered.  ○ Although the existence of major confounding variables was confirmed, these variables were not adequately considered during the design and analysis phases.  **Before-after study**  ○ Natural progression and a learning effect are relatively evident in the considerations of diseases and interventions. |
| Criteria for judgments of an 'Unclear risk' of bias | It is uncertain whether the incomplete outcome data resulted in a 'high risk' or a 'low risk' of bias. |
| * This effect occurs if past experience improves future execution skills. | |
| 3. Measurement of exposure  Performance biases caused by inadequate measurements of exposure | |
| Criteria for judgments of a 'Low risk' of bias | If exposure data were described using at least one of the methods that are listed below:  ○ Data were obtained from trustworthy sources, such as medical records.  ○ Data were obtained from structured interviews. |
| Criteria for judgments of a 'High risk' of bias | Any one of the following conditions:  ○ Data were obtained through self-reported methods  ○ A clear case of interviewer bias*  ○ A clear case of recall bias** |
| Criteria for judgments of an 'Unclear risk' of bias | It is uncertain whether the incomplete outcome data resulted in a 'high risk' or a 'low risk' of bias. |
| * “Interviewer bias” describes a situation in which the characteristics of the investigators cause the study data to be standardized in a manner that affects the study results. This phenomenon can be reduced through the training of investigators.  ** “Recall bias” describes a situation in which the respondents' degree of recall can affect the study results. | |
| 4. Blinding of outcome assessments  Detection biases caused by the inadequate blinding of outcome assessments | |
| Criteria for judgments of a 'Low risk' of bias | Any one of the following conditions:  ○ The outcome assessments were blinded  ○ Although blinding was not present, its absence was judged to have no effect on the outcome measurements. |
| Criteria for judgments of a 'High risk' of bias | Blinding was not performed or incomplete, and this lack of appropriate blinding appears likely to have affected the outcome measurements. |
| Criteria for judgments of an 'Unclear risk' of bias | It is uncertain whether the incomplete outcome data resulted in a 'high risk' or a 'low risk' of bias. |
| 5. Incomplete outcome data  Attrition biases caused by the inadequate handling of incomplete outcome data | |
| Criteria for judgments of a 'Low risk' of bias | Any one of the following conditions:  **Non-randomized studies (except for before-after studies)**  ○ There are no missing data.  ○ The causes of any missing data are considered to be relevant to the study outcomes (i.e., censoring does not create a bias in the survival data).  ○ The quantity of missing data was a product of similar developments in both the intervention (exposure) and the control groups, and the causes of these developments are similar.  **Before-after study**  ○ Information about the number of participants before and after the study exists, and the baseline did not differ with respect to completed and failed study participants. |
| Criteria for judgments of a 'High risk' of bias | Any one of the following conditions:  **Non-randomized studies (except for before-after studies)**  ○ The missing data could affect the study outcome. These effects may be attributed to the differences in the missing data between the intervention (exposure) group and the control group, or the effects may be caused by the absence of important measurements.  **Before-after study**  ○ Differences exist with respect to the baseline for successful and failed participants. |
| Criteria for judgments of an 'Unclear risk' of bias | It is uncertain whether the incomplete outcome data resulted in a 'high risk' or a 'low risk' of bias. |
| 6. Selective outcome reporting  Reporting biases caused by the selective reporting of outcomes | |
| Criteria for judgments of a 'Low risk' of bias | Any one of the following conditions:  ○ The experimental protocol is available, and the pre-defined primary/secondary outcomes were described as planned.  ○ All of the expected outcomes were included in the study descriptions (even in the absence of the experimental protocols). |
| Criteria for judgments of a 'High risk' of bias | Any one of the following conditions:  ○ The pre-defined primary outcomes were not fully reported.  ○ The outcomes were not reported in accordance with the previously defined standards.  ○ Primary outcomes that were not pre-specified in the study existed (except for outcomes with clear explanations, such as unexpected adverse effects).  ○ The existence of incomplete reporting regarding the primary outcome of interest.  ○ The absence of reports on important outcomes that would be expected to be reported for studies in related fields. |
| Criteria for judgments of an 'Unclear risk' of bias | It is uncertain whether the selective outcome reporting resulted in a 'high risk' or a 'low risk' of bias. |
